# Birdshot Chorioretinopathy in a Patient of Filipino Ancestry: A Case Report

**DOI:** 10.7759/cureus.100722

**Published:** 2026-01-04

**Authors:** Ikhlas Mahmoud, Joanna DaCosta

**Affiliations:** 1 Newcastle Eye Centre, Royal Victoria Infirmary, Newcastle, GBR

**Keywords:** bilateral vitritis, birdshot chorioretinopathy, filipino, hla-a29, retinal vasculitis

## Abstract

Birdshot chorioretinopathy (BCR) is an uncommon bilateral posterior uveitis, resulting in characteristic cream-coloured spots scattered across the fundus resembling the pattern of birdshot from a gun. Typically, the condition is diagnosed in middle-aged individuals of Northern European descent.

We report the case of a 48-year-old male Filipino patient with BCR. The presenting symptoms, examination findings, and investigations confirming the diagnosis are outlined. Treatment and subsequent clinical course are also described.

To our knowledge, this is the first report of BCR in a Filipino patient. The diagnosis should be considered in all patients based on clinical and investigative findings. Awareness that the disease is not limited to those of Northern European descent is important to avoid delays in diagnosis and institute treatment to preserve visual function.

## Introduction

Birdshot chorioretinopathy (BCR) is a chronic, bilateral posterior uveitis characterised by cream-coloured choroidal lesions in the fundus associated with vitritis, retinal venous vasculitis and, in some cases, optic disc swelling and cystoid macular oedema.

The condition is strongly associated with the HLA-A29 antigen, which is present in up to 98% of reported cases. However, the HLA-A29 antigen is also positive in 7% of the normal population [1]; therefore, it is not diagnostic in isolation, as many HLA-A29-positive individuals do not develop the disease. Consequently, HLA-A29 serves as an important supportive diagnostic criterion when interpreted in the appropriate clinical context.

The disease is most commonly described in Caucasians of Northern European ancestry, with only rare case reports in African American [2], Hispanic [3], Brazilian [4], South Asian [5] patients and, more recently, East Asian patients, including Japanese [6] and Chinese patients [7].

To our knowledge, this is the first reported case in a Filipino patient. The rarity of this condition in Southeast Asian populations may lead to delays in diagnosis, potentially resulting in irreversible retinal dysfunction or vision loss due to delayed initiation of treatment [1,8].

## Case presentation

**Figure 1:**
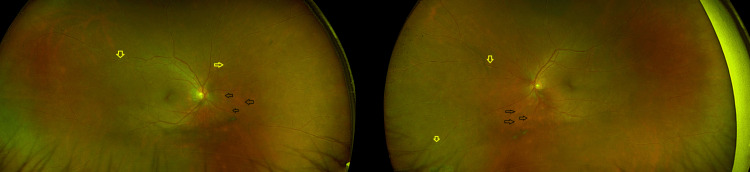
Widefield colour fundus photography of both eyes (right eye to the left-hand side and left eye to the right). Black-bordered arrows point towards some of the cream coloured oval choroidal lesions inferonasally. Yellow-bordered arrows point towards the vasculitis described.

A 48-year-old Filipino male presented with a total 18-month history of floaters and blurriness in both eyes. Initial presentation was to his local hospital after he was referred to them for having floaters a few months prior and a diagnosis of vitritis, and he was commenced on oral steroids. As his steroid dose was tapered, he became symptomatic, prompting referral to the tertiary uveitis centre at our department to consider immunosuppression; he presented to us after being under their care for 15 months. His past medical history included treated syphilis a decade earlier. He denied ocular pain, redness or photophobia. The systemic review was unrevealing for an association with uveitis. There was no history of tick bites or tattoos. He had a past left inferonasal retinopexy. On examination, his corrected visual acuity was 6/9 in the right eye and 6/6 in the left eye. No relative afferent pupillary defect was present. Ishihara colour vision was full in both eyes. Intraocular pressures were normal. The anterior segment in both eyes was normal with clear lenses. The posterior segment showed +2 vitreous cells and +1 vitreous haze in both eyes, with pale cream-coloured oval choroidal lesions in all quadrants, being most prominent inferonasally, and peripheral retinal venous vasculitis with no vitreous snowballs and no optic disc swelling (Figure 1).

**Figure 2:**
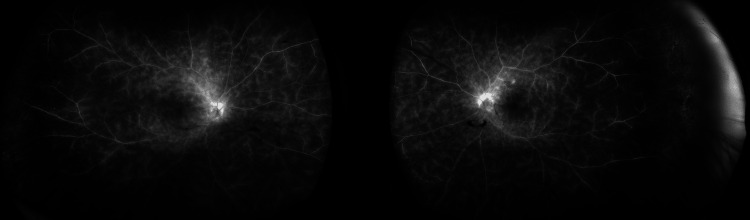
Widefield fluorescein angiography of both eyes (right eye to the left-hand side and left eye to the right), late venous phase depicting widespread leakage and optic disc staining

The clinical impression was of intermediate uveitis. Widefield fluorescein angiography (FA) demonstrated widespread retinal vascular leakage with no angiographic cystoid macular oedema (Figure 2).

**Figure 3:**
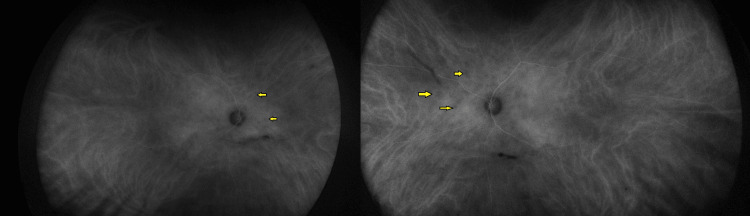
Widefield indocyanine green of both eyes (right eye to the left hand side and left eye to the right) Yellow arrows point to some of the hypofluorescent lesions nasally.

Indocyanine green (ICG) showed multiple hypofluorescent spots throughout the posterior pole and mid-periphery (Figure 3).

**Figure 4:**
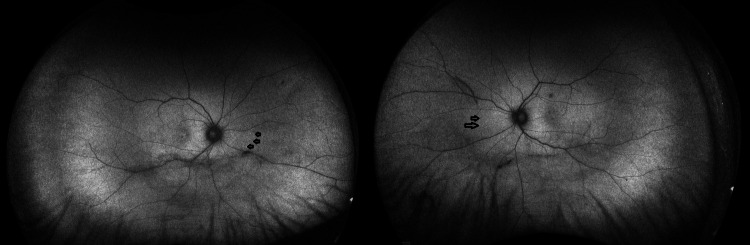
Widefield fundus autofluorescence of both eyes (right eye to the left hand side and left eye to the right). Black arrows indicate a few of the hypoautofluorescent spots nasally.

Fundus autofluorescence (FAF) revealed multiple hypoautoflurorescent lesions corresponding to clinical and ICG findings, with patchy hyperautofluorescent borders indicative of active disease (Figure 4).

**Figure 5:**
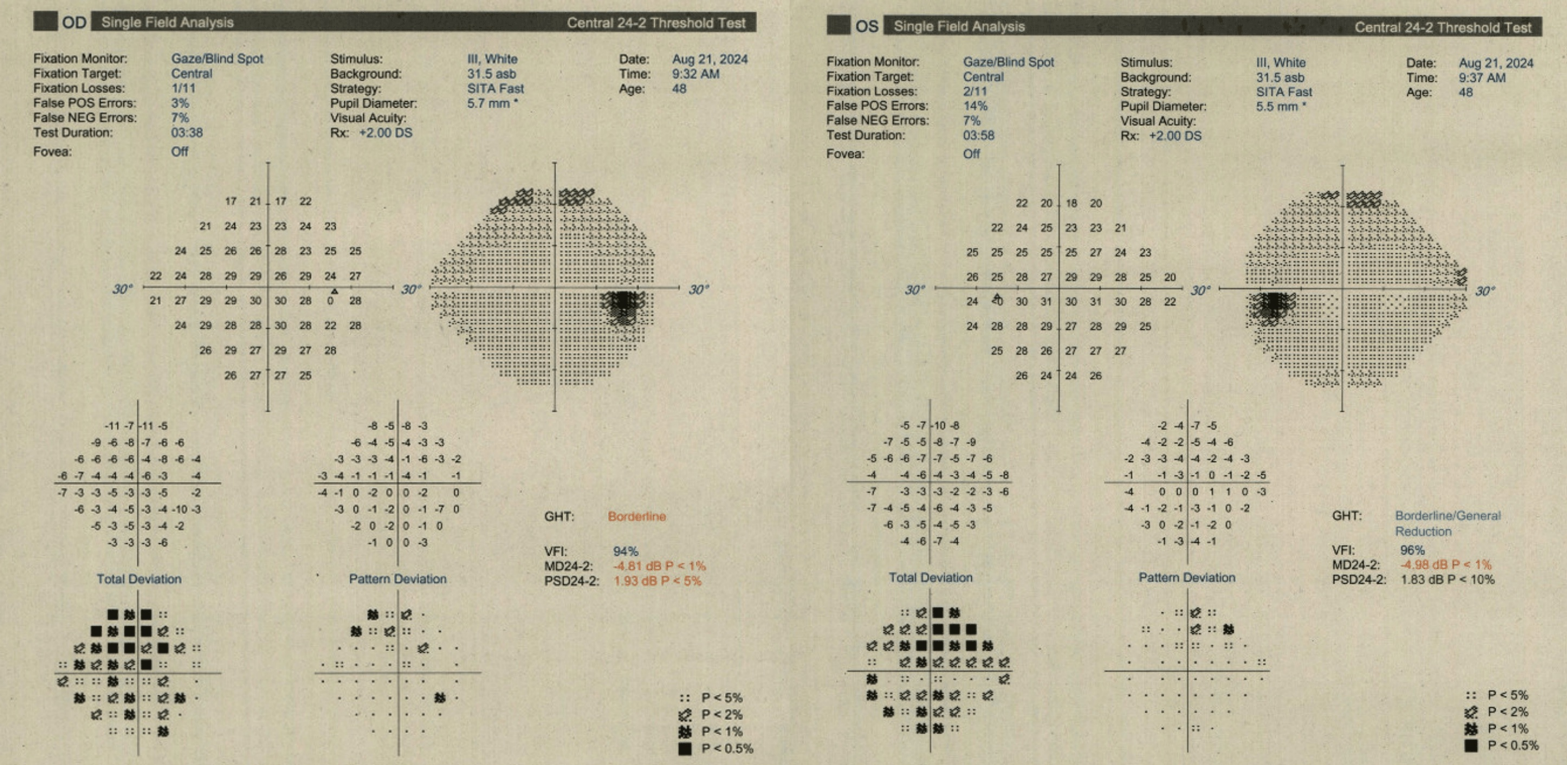
Visual field testing of both eyes using the 24-2 Swedish Interactive Threshold Algorithm (SITA) Fast strategy (right eye on the left-hand side and left eye on the right-hand side) showed mean deviations of -4.8 dB in the right eye and -4.9 dB in the left eye.

Optical coherence tomography (Heidelberg) of the macula showed no cystoid macular oedema. Humphrey visual fields (24-2) revealed bilateral generalised depression (Figure 5).

Blood tests, as shown in Table 1, revealed a negative QuantiFERON-TB Gold test and evidence of previous syphilis infection, indicated by a positive treponemal antibody test with negative rapid plasma reagin (RPR) and negative syphilis IgM enzyme-linked immunosorbent assay (ELISA). Tests for toxoplasmosis were negative. Hepatitis B core Ab was positive at presentation (polymerase chain reaction two months later showed it was not detectable), hepatitis B surface antigen and hepatitis B surface antibody were negative, and hepatitis B viral DNA was undetectable. The hepatitis C antibody was negative. HIV 1+2 antibody/p24 antigen was negative. Angiotensin-converting enzyme and HbA1c were normal. Computed tomography of the chest with contrast was normal with no hilar lymphadenopathy. Liver function tests, urea and electrolytes were within normal limits. The HLA-A29 antigen was positive. This extensive workup ruled out an infectious cause.

**Table 1:** Summary of laboratory investigations performed to rule out infectious causes Ag: antigen; Ab: antibodies; RPR: rapid plasma reagin; ELISA: enzyme-linked immunosorbent assay; ALP: alkaline phosphatase; ALT: alanine aminotransferase; ACE: angiotensin-converting enzyme

Test	Result	Reference Range
Quantiferon-TB Gold	Negative	
HIV 1+2 Ab / p24 Ag	Not detected	
Hepatitis B Core Ab	Detected at presentation, 2 months later undetectable by PCR	
Hepatitis B Surface Ab	Not detected	
Hepatitis B Surface Ag	Not detected	
Hepatitis C Ab	Not detected	
Treponemal Ab test	Detected	
RPR	Negative	
Syphilis IgM ELISA	Not detected	
Toxoplasma IgG	Not detected	
HLA-A29	Positive	
Sodium	142 mmol/L	133–146 mmol/L
Potassium	4.4 mmol/L	3.5–5.3 mmol/L
Urea	5.8 mmol/L	2.5–7.8 mmol/L
Creatinine	85 µmol/L	59–104 µmol/L
Total protein	71 g/L	60–80 g/L
Albumin	48 g/L	35–50 g/L
Corrected calcium	2.46 mmol/L	2.2–2.6 mmol/L
Phosphate	0.94 mmol/L	0.80–1.80 mmol/L
Bilirubin	8 µmol/L	0–21 µmol/L
ALP	58 U/L	30–130 U/L
ALT	20 U/L	0–40 U/L
HbA1c	38 mmol/mol	0–47 mmol/mol
ACE	40 U/L	20–70 U/L

His full-field electroretinogram showed generalised inner and outer retinal dysfunction in both eyes, more marked in the rod system. Multifocal electroretinogram revealed significant macular dysfunction in both eyes.

All the above led to the diagnosis of BCR. He was advised to continue prednisolone 10 mg daily and was initially reviewed every two to four weeks. As persistent vitritis was noted, mycophenolate mofetil was commenced at 500 mg twice daily and subsequently escalated to 1.5 g twice daily after confirming the absence of adverse effects.

**Figure 6:**
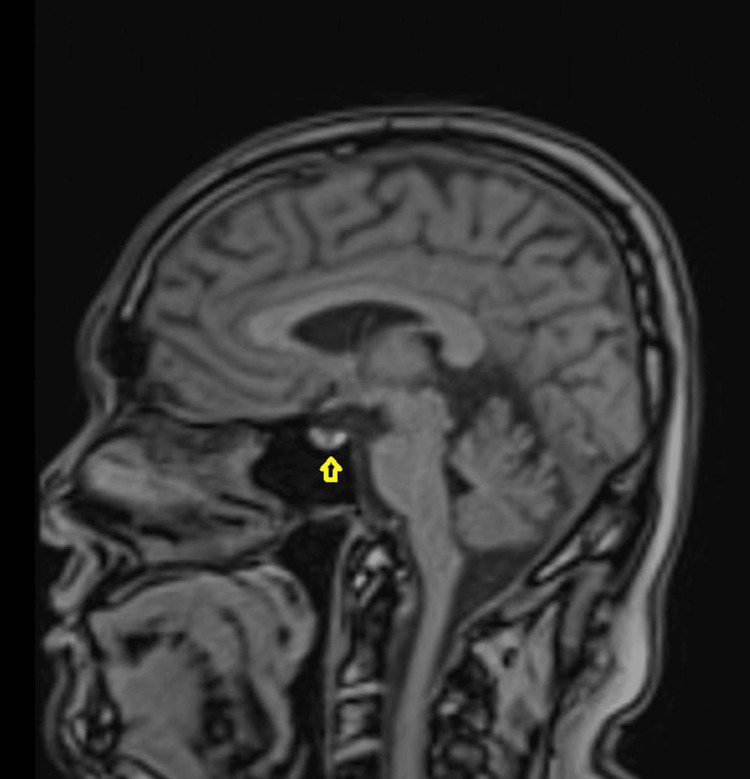
Sagittal T1-weighted MRI of the brain. The yellow arrow depicts the pituitary adenoma.

As the patient continued to have +1 vitritis, a brain MRI with contrast was performed prior to the planned addition of adalimumab. This demonstrated no evidence of demyelination but revealed an incidental pituitary microadenoma (Figure 6), for which an endocrinology opinion was requested.

At the most recent visit, the patient was on 6 mg oral prednisolone and 1.5 g mycophenolate mofetil twice daily, and his visual acuity remained stable at 6/9 in the right eye and improved to 6/6 in the left eye, with persistent +1 vitritis bilaterally. The patient reported symptomatic improvement. Repeat electrodiagnostic testing is planned, and the patient is awaiting endocrinology review.

## Discussion

The patient described was of Filipino ancestry with a bilateral intermediate uveitis and HLA-A29 positive. Other investigations were unrevealing as to the underlying aetiology; hence, the diagnosis of BCR. Several case reports have highlighted that the clinical features of BCR in patients not of Caucasian descent often resemble those in Caucasian patients, though with some variations. Knezevic et al. [2] described an African American woman with typical creamy choroidal lesions and vitritis, while Baddar and Goldstein [3] reported a Hispanic patient with the characteristic lesions but also disc leakage and cystoid macular oedema. In the Brazilian multicentre review, da Fonsêca et al. [4] observed that most patients exhibited the classic fundus lesions, though in those with more pigmented fundi, the spots were less apparent. Apivatthakakul et al. [5] documented a South Asian patient who presented with classical lesions but more prominent macular involvement, including oedema. Similarly, Saito et al. [6] described the case of a Japanese patient with typical creamy lesions and vasculitis, and Regenold et al. [7] reported a Chinese patient whose fundus demonstrated the classical features. Our Filipino patient displayed more prominent inferonasal creamy lesions consistent with these prior observations, though, as in the Brazilian cohort [4], visibility may have been influenced by fundus pigmentation. FA has shown remarkable consistency across ethnicities. Knezevic et al. [2], Baddar and Goldstein [3], da Fonsêca [4], Apivatthakakul et al. [5], Saito et al. [6], and Regenold et al. [7] each documented diffuse vascular leakage, sometimes accompanied by disc staining or macular oedema. Our patient’s FA similarly demonstrated widespread leakage without cystoid macular oedema, aligning with these reports and suggesting that angiographic findings are largely uniform across populations. ICG angiography has likewise been a reliable tool across case series, consistently demonstrating a higher burden of disease than is clinically evident. da Fonsêca et al. [4] described widespread hyperfluorescent lesions in Brazilian patients, while both Saito et al. [6] and Regenold et al. [7] observed numerous dark dots extending beyond the clinically visible lesions in Japanese and Chinese patients, respectively. Our patient’s ICG findings mirrored this pattern, reinforcing the role of ICG in identifying subclinical choroidal involvement across ethnic backgrounds. Electrophysiological testing has also shown concordant abnormalities in different populations. Da Fonsêca et al. [4] reported diffuse electroretinography (ERG) abnormalities in most of their Brazilian patients, and Regenold et al. [7] documented rod and cone dysfunction in a Chinese patient. Our patient’s ERG demonstrated generalised inner and outer retinal dysfunction with rod-predominant involvement, together with macular dysfunction on multifocal ERG, findings that are consistent with those reported in Caucasian cohorts [1] as well as in patients of African American [2], Hispanic [3], Brazilian [4], South Asian [5], Japanese [6], and Chinese ancestry [7]. A recurring theme across reports in African American [2] and Brazilian patients [4] is diagnostic delay, attributable in part to lower clinical suspicion and to reduced visibility of fundus lesions in more heavily pigmented eyes. This underscores the need for multimodal imaging and electrophysiology to support early diagnosis regardless of ancestry.

Finally, early initiation of immunomodulatory therapy is critical for preserving visual function. Silpa-Archa et al. [8] identified delayed immunosuppression as a poor prognostic factor, and consensus diagnostic criteria have reinforced the importance of prompt recognition and treatment as described by Levison et al. [9]. In our patient, the early introduction and escalation of mycophenolate mofetil, with a plan to add biologic therapy if required, reflects best practice and highlights the importance of aggressive management in sustaining vision.

## Conclusions

This report describes a case of BCR in a Filipino patient, adding to the limited literature on the occurrence of this condition outside its traditionally recognised demographic groups. Although BCR is most frequently reported in individuals of Northern European ancestry, this case demonstrates that the disease can present with typical clinical, angiographic, and electrophysiological features in patients from Southeast Asian backgrounds. Recognition of these features remains critical, regardless of ethnicity.

Given the rarity of BCR in more pigmented populations, there is a risk of delayed diagnosis, particularly when fundus lesions are less conspicuous. Multimodal imaging and electrophysiological testing play an important role in supporting the diagnosis and assessing disease activity in such cases. Early identification and timely initiation of immunosuppressive therapy are essential to limit cumulative retinal damage and preserve visual function. This case reinforces the need for heightened clinical awareness of BCR across diverse ethnic populations.
